# Cross-cultural adaptation and validation of the Arabic version of the knee and hip health-related quality of life (Mini-OAKHQOL) questionnaire in male Saudi patients with osteoarthritis: a methodological observational design

**DOI:** 10.7717/peerj.18122

**Published:** 2024-09-25

**Authors:** Madi Talal Alharbi, Mahamed Ateef, Ahmad Alanazi, Msaad Alzhrani

**Affiliations:** 1 Department of Physiotherapy, Sabya General Hospital, Sabya, Jazan Region, Saudi Arabia; 2 Department of Physical Therapy and Health Rehabilitation, College of Applied Medical Sciences, Majmaah University, Majmaah, Saudi Arabia

**Keywords:** Osteoarthritis, Knee, Hip, Quality of life, Arabic, Reliability, Validity, Scale

## Abstract

**Background:**

Osteoarthritis (OA) is common in Saudi Arabia, has a significant impact on quality of life (QoL), and lacks a specific questionnaire to measure QoL. The primary objective of this study was to translate and cross-culturally adapt the Mini Osteoarthritis Knee and Hip Quality of Life (Mini-OAKHQOL) questionnaire into Arabic and to determine its psychometric properties among OA knee and/or hip patients in Saudi Arabia.

**Methods:**

A methodological observational design was conducted and followed standard guidelines for cross-cultural adaptation of Mini-OAKHQOL into Saudi Arabic. Two hundred and eight primary OA knee and/or hip male participants aged between 45 and 80 years with a mean age of 58.65 ± 13.8 years and a BMI of 29.5 ± 1.2 kg/m^2^ were included and performed the stages of translation to target Arabic language (forward T1 and T2), synthesized an Arabic draft (T12), then back-translated to English (BT1 and BT2), followed by expert committee review to rectify the deficiencies leading to a prefinal stage involving a pilot test on native Arabic speakers, thereby finalized a final Arabic version. The Arabic Mini-OAKHQOL, Arabic Short Form 12 (SF12), and visual analog scale (VAS) were administered to analyze internal consistency (IC), test–retest reliability at baseline as well as one week later (up to the 10^th^ day). The construct validity was tested using Spearman’s rank correlation, and factor analysis was done to establish a five-factor fit model. Homogeneity was determined using principal component analysis (PCA). Floor and ceiling effects calculated in percentages.

**Results:**

The Arabic Mini-OAKHQOL showed an excellent Cronbach’s alpha of the overall scale (*α* = 0.931) for its internal consistency and an excellent intraclass correlation coefficient (ICC) of 0.947 for its retest reliability, with a high response rate of 93.75%. The construct validity of this scale was good with Ar-SF12 and VAS pain. A five-factor model fit was considered acceptable, and factor loading for each item found within the permissible limits confirmed the factorial validity. None of the items, dimensions, or overall scale showed either a floor or ceiling effect.

**Conclusion:**

The adapted and tested Arabic Mini-OAKHQOL is a reliable and valid questionnaire to measure the impact of knee and/or hip OA on quality of life in the Saudi Arabian male OA population to reduce the respondent’s burden for use in clinical and prospective studies.

## Introduction

The most common cause of disability among the elderly is osteoarthritis (OA) (Kiadaliri et al., 2018). It tops the list of chronic diseases and is a concern for public health (Hawker, 2019), and over 250 million individuals worldwide suffer from arthritis, with OA knee being the most common (Messier et al., 2021). The main effects of OA are a decrease in quality of life (QoL) due to chronic pain and physical disability (Chen, Huang & Liao, 2023). The prevalence of OA increases with aging, and is more predominant in women. According to the World Health Organization (WHO) 2023 study, OA commonly appears in the late 40s to mid-50s, and 73% of participants are older than 55 years, and 60% of them are female. OA incidence is expected to rise by 74.9% for the knee, 48.6% for the hand, 78.6% for the hip, and 95.1% for other joints by 2050 as compared to 2020, with the knee being the overall burden and the hip having the highest annual percentage change (APC) (Long et al., 2022; WHO, 2023). According to the Global Burden of Disease 2019 study, the prevalence of OA was 10.5% in the Middle East between the ages of 55 and 59 years; the highest among women in the various age ranges was not substantially greater than that of men, and knee OA is the most common, followed by hip OA (Shamekh et al., 2022).

Recent studies have reported the prevalence of OA knee in Saudi Arabia (SA) at an overall 18.9%, with females at 20.3% and males at 13.1% (Althomali et al., 2023) and hip OA at 4.1% (Aldhilan et al., 2022). Significant issues such as severity of pain (Bindawas et al., 2018), modified prayer position (Ateef et al., 2021), comorbidities (Bashekah et al., 2023), age, low literacy, body mass index (BMI), and floor sitting are associated with OA and are linked to poor physical and mental health and declined QoL in SA (Vennu et al., 2023). Reducing the burden of this disease in Saudi might be achieved by efforts to educate the public and lifestyle changes (Al-Hazzaa, 2018; Makhdom & Bokhary, 2023). A study of 480 OA knee participants, mostly aged 40–45 and older than 65 years, reported a high prevalence rate of 77.8% of knee pain and a decline in QoL in the Jazan region of SA (Zenat Khired et al., 2023). The general well-being of people in societies could be measured using the concept of QoL. The World Health Organization explains QoL as a subjective evaluation of one’s perception of their reality relative to their goals as observed through their culture and value system. Another subset of QoL is health-related quality of life (HRQOL), a multi-dimensional concept commonly used to examine the impact of any health status on QoL. Making decisions in clinical practice may be aided by this kind of information, and members of the healthcare system need to recognize the concept of QoL (Teoli & Bhardwaj, 2023).

Tools used to measure patient-reported outcomes (PROs) are called patient-reported outcome measures (PROMs). These tools are self-completed surveys by the participants to assess their health status. Measures of functional status, health-related quality of life, symptom stress and intensity, individual experiences with treatment, and health-related behavior may all be assessed using PROMs. They are generic or disease-specific tools (Churruca et al., 2021). Generic tools are used to assess the age, health status, and QoL of healthy and unhealthy individuals and treatment groups, whereas disease-specific tools identify the biological and psychological components of a specific disease and its impact on QoL. Assessment of PROMs is an emerging topic, and a review emphasizes the increasing awareness on a global basis that including the patient’s perspective is essential to the quality of healthcare (Nelson et al., 2015; Churruca et al., 2021). A systematic review of different PROMs has analysed for their methodological qualities (Ateef et al., 2022), and PROMs could more closely match health care delivery with patient priorities, but only with careful selection (Liu, Rothrock & Edelen, 2024).

Knee OA significantly impairs functioning and lowers an individual’s QoL as well as socioeconomic standing (Mahmoud et al., 2019). High costs associated with therapy, frequent medical visits, and hospital stays will have a detrimental effect on QoL. Health care practitioners can consider patients’ perceptions of their own health status because of the availability of measurement tools, and making decisions in clinical practice may be aided by this kind of information (De Bienassis et al., 2021). Generic Short-Form 12 (SF 12) and World Health Organization Quality of Life (WHOQOL) scales are frequently used to measure general QoL among healthy and unhealthy individuals but are not specific to disease-related QoL (Soylu & Kütük, 2022). The Western Ontario and McMaster Universities Osteoarthritis (WOMAC) questionnaires can only evaluate pain and function, and they cannot measure areas of QoL, such as mental, social, and sexual domains (Alghadir et al., 2016; Tuncay Duruöz et al., 2022). Knee injury and Osteoarthritis Outcome Score (KOOS) has one domain to measure QoL but not related hip (Ateef et al., 2022). Knee Injury and Osteoarthritis Outcome Score Patello-Femoral (KOOS-PF) questionnaire has only one item related to QoL (Ateef, 2020). The Osteoarthritis Knee and Hip Quality of Life (OAKHQOL) questionnaire was developed to measure the effect of knee and hip OA on QoL (Rat et al., 2005; Tuncay Duruöz et al., 2022). The long OAKHQOL has 43 items, 40 of which fall into five categories: physical activity, mental health, pain, social support, and social functioning. The remaining three are independent items. The first version demonstrated psychometric qualities required for use in research (Rat et al., 2005; Rat et al., 2006). There have been several translated versions of the original OAKHQOL questionnaire, both in long and short versions. A 43-item-long version has been translated into Spanish, Moroccan Arabic, Percian, Chinese, and Hungarian. A 20-item short version has been translated into Spanish and Turkish. The Mini-OAKHQOL has demonstrated face and content validity based on its International Classification of Functioning (ICF) core set of dimensions and has an excellent reliability and construct validity to measure the impact of OA knee and hip on QoL (Guillemin et al., 2016).

A recent study in Saudi Arabia evaluated the QoL using the Ar-SF-36 generic questionnaire among OA knee participants and found that knee OA had considerably lowered HRQoL (Bindawas et al., 2018). Using a disease-specific scale, clinicians can track QoL at early stages, thereby enabling preventive strategies to be adapted to enhance QoL at late stages. Saudi Arabia is experiencing a rise in OA and its burden (Shamekh et al., 2022). According to a systematic review conducted in 2020, there have been four HRQoL scales validated in standard Arabic in Saudi Arabia: two are generic, one specific, and the other one is in a mixed population (Al Maqbali et al., 2020). One disease-specific QoL Arabic questionnaire, Osteoarthritis Quality of Life questionnaire (OAQoL), was translated and validated in Saudi OA population. It is meant to evaluate the general OA impact on QoL but is not specific to knee or hip OA and has methodological weaknesses such as inadequate sample size and did not measure test–retest reliability (AlAjmi & Al-Ghamdi, 2021). Along with physical activity, OA also influences mental health and social functioning, thereby declining QoL (Lopes et al., 2023). Disease-specific Mini OAKHQOL captures these factors that are associated with osteoarthritis of the knee and hip (Rat et al., 2005; Rat et al., 2006; Guillemin et al., 2016). Therefore, Saudi Arabia needs questionnaires to assess how knee or hip OA affects these factors in the Saudi population and, thereby, benefits the patient-centered treatment strategies (Bashekah et al., 2023).

The reason for adapting Mini-OAKHQOL in the Saudi OA population was that the Moroccan Arabic long form of OAKHQOL has 43 items (Serhier et al., 2012), which would be a burden to the participants in any research setup (Sharma, 2022). Moroccan Arabic is significantly different from Modern Standard Arabic (MSA), and Saudi Arabic is MSA. The Moroccan version is a Moroccan dialect, and it differs noticeably from most other Arabic languages in terms of vocabulary, syntax, grammar, and pronunciation. Moreover, 10% to 15% of its vocabulary is made up of loanwords from the Berber language, which are mixed with French and Spanish (Wexler, 2012) and are difficult for Saudis to read and use. Moreover, the mini scale has benefits over long form in reducing time and funds, as well as producing fewer missing data and respondent denials (Guillemin et al., 2016). The short form has evolved from the long form, OAKHQOL, with its refined methodological quality and availability in the literature; hence, it needed to be validated in other languages to fill the literature gap and to significantly increase the response rate using short scales over long scales in the research studies (Guillemin et al., 2016; Sharma, 2022). Therefore, the aim of this study was to translate and cross-culturally adapt the Mini-OAKHQOL questionnaire into Arabic and validate its psychometric properties among the OA knee and/or hip population to reduce the respondents’ burden in Saudi Arabia.

## Materials & Methods

### Study design and setting

A methodological research study of observational design was conducted at two Ministry of Health (MOH) hospitals and 69 primary health care centers (PHC) for cross-cultural adaptation of the Mini-OAKHQOL questionnaire. PROMs such as disease-specific Ar-Mini-OAKHQOL and generic Ar-SF-12 were used to assess the QoL of knee and hip OA patients in Jazan region.

#### Ethical approval

Written permission was obtained from the original developer of the Mini-OAKHQOL questionnaire to translate and culturally adapt it into Saudi Arabic by the copyright holders (Guillemin et al., 2016). The protocol was approved prior to the start of the study by the Jazan Health Ethics Committee of the Ministry of Health of the Kingdom of Saudi Arabia (KSA) (NO: 2247).

### Sample size

According to the recommendations for questionnaire validation, based on a rule of thumb, 10 samples would be required for each item (respondent-to-item ratio of 1:10). The Mini-OAKHQOL has 20 items, *i.e.,* 20 × 10 = 200 (*n* = 200) (Bujang et al., 2012; Tsang, Royse & Terkawi, 2017; Bujang et al., 2019; Memon et al., 2020; Gunawan, Marzilli & Aungsuroch, 2021; Bujang et al., 2024). Keeping in view the dropout numbers, we have taken an extra eight samples.

### Patient demographics

Two hundred and eight male patients aged between 45 and 80 years as a common prevalent age range for primary OA of the knee and/or the hip (Zenat Khired et al., 2023; Vennu et al., 2023) whose mean age was 58.65 ± 13.8 years and BMI was 29.5 ± 1.2 kg/m^2^ and diagnosed by orthopedic surgeons with the ability to read and write Saudi Arabic were included. We received the participants after the diagnosis, medical aspects of the exclusion criteria were excluded by the orthopedic surgeons, and we excluded participants who were unable to read and write Arabic, had eye vision issues, had a history of joint replacement (Wang et al., 2016), had poor mental abilities to understand the Arabic, were unwilling to provide written informed consent, and were non-Saudis. The authors used only the male participants in this study, as the male physical therapists as evaluators were not allowed to treat or evaluate female participants in the hospitals in Saudi Arabia.

### The patient reported outcome measures

### Description of the mini-OAKHQOL questionnaire

The Mini-OAKHQOL, a condensed version of the long 43-item OAKHQOL that was recently developed, has demonstrated excellent validity and reliability. It maintained the same structure and 20 items from the original instrument, with the five dimensions of physical activities (seven items), mental health (three items), pain (three items), social support (two items), and social functioning (two items), as well as three independent items addressing sexual life, professional life, and fear of dependency. It takes 10–15 min to fill. The scoring system includes numerical rating scales ranging from 0 (worst) to 10 (best). The means of the item scores in each subscale are calculated to determine the scores. The mean item score for dimensions. The subscales measuring social functioning and social support are rated similarly to the “0 (worst)-10 (best),” although they have opposite questions. The equivalent score is determined by adding the scores of the final three independent items (Guillemin et al., 2016; Tuncay Duruöz et al., 2022).

### Other outcome measures

SF12 is a generic measure that evaluates quality of life, and a condensed form of SF36 has fewer items than other questionnaires. There are 12 items in it, divided into eight subdimensions: social functioning (1 item), role-emotional (2 items), physical functioning (2 items), bodily pain (1 item), general health (1 item), energy (1 item), and mental health (2 items). The other items are Likert-type, with response options ranging from 3 to 6, whereas the questions on role-physical and role-emotional are dichotomous, to be answered as “yes or no.” The subdimensions on general health, physical functioning, role-physical, and bodily pain yield the total PCS-12 score, whereas the subdimensions on social functioning, role-emotional, mental health, and energy yield the total MCS-12 score. Higher scores indicate a better health condition. The PCS-12 and MCS-12 scores both range from 0 to 100 (Haddad et al., 2021; Soylu & Kütük, 2022).

### Cross-cultural adaptation protocol

The process described by Beaton et al. (2000) was applied to the translation and cross-cultural adaptation of Ar-Mini-OAKHQOL. The five stages are forward translation, synthesis, backward translation, expert committee review, and prefinal testing.

### Stage I: translation (forward translation)

The original Mini-OAKHQOL English version was translated (forward translation) into the targeted Arabic language by two separate bilingual native Arabic translators. The first translator was a senior physiotherapist for clinical equivalency (T1-informed) who was proficient in both Arabic, English, and the subject. A non-medical second blinded translator (T2-uninformed) was a native Arabic and fluent English reader, and then both translated the original Mini-OAKHQOL into Arabic as separate translations. This stage allowed for a comparison of the translations and the noting of the differences that could indicate a more unclear original language or differences in the translation process. In an exchange of ideas, the translators pinpoint and fix poor language decisions. Every translator completes a written report detailing their translation work.

### Stage II: synthesis of the translations

Based on the results of the translated versions of T1 and T2, a synthesized version was extracted as draft T12 with a documented report. The translators discussed the content of both versions, addressed any discrepancies between them, and ultimately produced a common Arabic draft, Mini-OAKHQOL (T12), with a moderator who was our department’s non-medical administrator.

### Stage III: back translation

A back translator (BT1) who is a Saudi native and an English professor by profession graduated from an overseas university, and a second back translator (BT2) who is also a Saudi native and an English teacher by profession graduated from an overseas university and is working in the local school, and both translated T12 back to its original English versions separately and were blinded to reduce the possibility of bias during the back translation process. This stage was meant for the validity of the translated version back to the original one for similarity in its items. Each translator produced a brief report after the translation.

### Stage IV: expert committee review

A total of four translators (two forward and two back translators), a developer, a lead researcher as a methodologist, an orthopeadician as a subject expert, and one native Arabic language professor made up the expert review committee. They convened to discuss and form a consensus report from all translated versions for semantic, idiomatic, experiential, and conceptual equivalence for their ease of reading, then for approval to produce a prefinal Arabic Mini-OAKHQOL version.

### Stage V: test of the prefinal Arabic Mini-OAKHQOL version

To validate a comprehensive Mini-OAKHQOL Arabic questionnaire and to collect feedback, a pilot test was conducted before the final version on 30 participants with a history of knee and hip OA, and these 30 participants were not included in the study and analysis. Pilot testing showed that the questions were clear and simpler without any additions or deletions confirming their face and content validity, and then a final Arabic Mini-OAKHQOL version was formed as final.

### Data collection

Participants provided a written informed consent form approved by the Ministry of Health, Jazan, Saudi Arabia, before participation. The convenience sampling method was used at two hospitals, the department of physiotherapy at King Fahad Central Hospital and Sabya General Hospital, along with 69 PHCs that are affiliated with these two MOH hospitals, between August 2022 and January 2023 in the Jazan region of Saudi Arabia, where the data was also collected from these PHCs to present a comprehensive range of clinical features and QoL of varied OA knee and hip patients. All participants were asked to provide demographic and clinical data. They also completed the Arabic Mini-OAKHQOL questionnaire along with the Arabic SF12 and VAS for pain intensity for construct validity. To establish the test–retest reliability, Arabic Mini-OAKHQOL was readministered after a minimum period of one week (Peasgood, Caruana & Mukuria, 2023) (up to 10th day) to prevent the effect of medication or physical therapy influence on the second measure. A total sample of 208 participants were screened as per the determined sample size for this study, and there were no dropouts during filling out the questionnaire or incomplete questionnaires for the estimated sample size of 208 (100%), but out of 208, thirteen respondents (6.25%) did not show up for the second session; 195 (93.75%) responded for the second session with a minimal attrition rate, which is acceptable; moreover, a minimum of 100 respondents is sufficient to conduct a test–retest reliability (Kennedy, 2022). This study had a surplus sample size for retest reliability, which demonstrates that the Arabic Mini-OAKHQOL is a reliable questionnaire.

### Measurement properties

#### Internal consistency

The Cronbach’s alpha measurement statistical test was used to assess the internal consistency of each questionnaire item. An item will be deleted if its value is less than 0.7, rated excellent if it is more than 0.9, and a good IC will be measured between 0.7 and 0.9 (Juul et al., 2016).

### Test–retest reliability

The reliability of the first and second (test–retest) assessments was compared using the intraclass correlation coefficient (ICC), with a 95% confidence interval. The ICC was calculated using an absolute agreement and the two-way random effect model. For good test–retest reliability, the ICC value should be greater than 0.80 (Juul et al., 2016).

### Construct validity

A minimum sample size is required to demonstrate construct validity, and the following formula is used: N = [(Z *α*+Z *β*)/C]^2^ + 3; where *C* = 0.5∗ln[(1 + *r*)/(1 − *r*)] = 0.3205, where *r* = 0.31 (expected construct validity); Z *α* = 0.05 = 1.96 (1 percent type I error rate, 10 percent type II error rate), Z *β* = 0.02 (Warren, 2023).

### Factorial validity

The 20-item Arabic Mini-OAKHQOL’s homogeneity was found using principal component analysis. Estimating sample size was the first stage in the factor analysis technique. Depending on how many items were in the questionnaire, a minimum sample size was needed for factor analysis. The factor analysis required 33–220 samples, with 3–20 samples needed for each item, per the recommendations (Memon et al., 2020). The Kaiser-Meyer-Olkin (KMO) measure was used to determine whether the sampling was adequate (Soriano-Alcantara, Guillén-Gámez & Ruiz-Palmero, 2024). The metric classified values between 0.5 and 0.8 as meritorious. The above elements were found to be fit to operate exploratory factor analysis (EFA) using Bartlett’s test of sphericity. The number of factors needed to demonstrate factorial validity was calculated using a scree plot with an eigenvalue >1 acting as the basis for the factor solution. The communalities produced by factor loading should be at least 0.3 (acceptable), 0.5 (moderate), and 0.8 (high). The minimum level of variance deemed acceptable to explain total item variance was 60 percent (Memon et al., 2020).

### Floor and ceiling effect

When less than 15% of the participants report having the lowest or highest possible score, the results are statistically acceptable. Each individual subscale’s ceiling (highest 100%) and floor (lowest 0%) effects have been noted separately in percentages (Juul et al., 2016).

### Data analysis

The demographic characteristics of the participant’s age and clinical characteristics were described in frequency and percentage for height, weight, and BMI in mean with standard deviation (SD) and 95% confidence interval (CI). Scale outcome measures were assessed for their normality using the Kolmogorov–Smirnov test. As the data did not follow a normal distribution, they were expressed with a median and an interquartile range. The internal consistency of the items was analysed by Cronbach’s alpha coefficient. Test–retest reliability was ascertained by the intraclass correlation coefficient (ICC). Factorial validity was performed with all the required parameters to establish construct validity, and Arabic Mini-OAKHQOL outcomes were correlated with Arabic SF12 outcomes and with VAS pain intensity using Spearman’s rank correlation coefficient. Finally, floor and ceiling effects were obtained as percentages. For all statistical analyses, the Statistical Package for Social Sciences (SPSS) software, Version 26, was utilized. The level of significance was fixed at 0.05.

### Results

#### Demographic and clinical characteristics

Male participants (*n* = 208) age ranged between 45 and 49 years were 66 participants (31.7%) and remaining above 49 years, and sample mean age was 58.65 ± 13.8 years, weight was 76.4 ± 15.6 ± 15.6 kg, height was 159.4 ± 12.8 cm, body mass index (BMI) 29.5 ± 1.2 kg/m^2^, knee OA was 88.47% (*n* = 184) and hip OA was 6.73% (*n* = 14) and mixed was 4.8% (*n* = 10) and presented in Table 1.

**Table 1:** Demographic and clinical characteristics of the participants (*n*= 208).

**Demographic dimensions**	**Frequency or Number (n)** **Mean, Standard Deviation (SD)**	**Percentage (%) /** **95% Confidence Interval (CI)**
Age (years)		
45 to 49	66	31.7
50 to 54	33	15.9
55 to 59	23	11.1
60 to 64	27	13
65 to 69	33	15.9
70 to 74	16	7.7
75 to 80	10	4.8
45–80	58.65 ± 13.8	45.5–79.8
Weight (kg)	76.4 ± 15.6	74.3 to 78.6
Height (cm)	159.4 ± 12.8	158.3 to 161.9
Body Mass Index (Kg/m2)	29.5 ± 1.2	28.9 to 32.1
**Osteoarthritis affected joint (s)**		
Left knee	40	19.2
Right knee	46	22.1
Bilateral knee	98	47.1
Left hip	6	2.9
Right hip	8	3.8
Bilateral hip and right knee	1	0.5
Left hip and bilateral knee	2	1
Left hip and right knee	2	1
Bilateral hip	1	0.5
Bilateral hip and bilateral knee	1	0.5
Left hip and left knee	2	1
Right hip and right knee	1	0.5

#### Translation and cross-cultural adaptation

During stage I process, only grammatical errors were encountered and rectified; during the stage II process, a few items were rephrased for a better flow of the native language and its readability. During stage III, dissimilarities have not been found in the back-translated items for their validity and during stage 4, semantic, idiomatic, experiential (target culture meanings), and conceptual (theoretical wordings) equivalences were not encountered.

#### Reliability

Overall Arabic Mini-OAKHQOL scale showed excellent reliability of internal consistency (*α* = 0.931) as shown in Table 2, while good to excellent IC for five individual dimensions ranged from *α* = 0.782 to 0.956 as shown in Table 3, and IC of all items as shown in Table 4.

**Table 2:** Demographic and clinical characteristics of the participants (*n* = 208). Reliability of Ar-Mini-OAKHQOL.

Ar-Mini-OAKHQOL dimensions	1st session (*n* = 208)	2nd session (*n* = 195)	Cronbach’s alpha (*α*)	ICC	ICC (95% CI)
Overall	51.3 (40.1, 61.2)	52.1 (40.4, 61.3)	0.931	0.947	0.774 to 0.983

**Table 3:** Test and retest reliability of Arabic Mini-OAKHQOL.

Ar-Mini-OAKHQOL dimensions	1st session (*n*= 208)	2nd session (*n*= 195)	Cronbach’s alpha (*α*)	ICC	ICC (95% CI)
Physical activity	47.1 (37.1, 62.9)	48.6 (37.1, 64.3)	0.942	0.968	0.821 to 0.983
Mental health	50 (36.7, 70)	50 (36.7, 70)	0.933	0.947	0.818 to 0.978
Pain	53.3 (36.7, 73.3)	56.7 (40. 73.3)	0.956	0.965	0.846 to 0.980
Social support	60 (50, 80)	60 (50, 80)	0.782	0.807	0.774 to 0.891
Social functioning	45 (25, 65)	50 (30, 65)	0.795	0.818	0.776 to 0.898
Independent items	43.3 (24.2, 60)	43.3 (26.7, 60)	0.877	0.915	0.937 to 0.944

**Notes.**

Ar-Mini-OAKHQOL Mini-Osteoarthritis Knee and Hip Quality of Life

ICC values are <0.5, indicated as poor reliability, between 0.5–0.75 indicates moderate reliability, between 0.75 and 0.90 explains good reliability and more than 0.90 is excellent reliability.

**Table 4:** Median, interquartile range of Internal consistency & test & retest reliability of all items.

S No	Ar-Mini-OAKHQOL items	1st session (*n*= 208)	2nd session (*n*= 195)	Cronbach’s alpha (*α*)	ICC (95% CI)
1	Q1-OAKHQOL	5 (3, 6.8)	5 (3, 7)	0.941	0.958 (0.954 to 0.961)
2	Q2-OAKHQOL	6 (3, 8)	6 (3, 8)	0.944	0.964 (0.959 to 0.968)
3	Q3-OAKHQOL	7 (4, 8)	7 (4, 8)	0.946	0.966 (0.962 to 0.969)
4	Q4-OAKHQOL	2 (0, 5)	2 (0, 5)	0.938	0.953 (0.945 to 0.964)
5	Q5-OAKHQOL	4 (2, 6)	4 (2, 6)	0.940	0.947 (0.943 to 0.957)
6	Q6-OAKHQOL	4.5 (2, 6)	4 (2, 7)	0.936	0.947 (0.941 to 0.964)
7	Q7-OAKHQOL	5 (2, 7)	5 (2, 7)	0.938	0.944 (0.931 to 0.950)
8	Q8-OAKHQOL	6 (3, 8)	6 (3, 8)	0.945	0.949 (0.941 to 0.959)
9	Q9-OAKHQOL	5 (2, 7)	5 (2, 7)	0.935	0.948 (0.941 to 0.962)
10	Q10-OAKHQOL	4 (0, 6)	4 (0, 6)	0.965	0.970 (0.961 to 0.974)
11	Q11-OAKHQOL	7 (5, 9)	7 (5, 9)	0.948	0.961 (0.957 to 0.978)
12	Q12-OAKHQOL	6 (4, 8)	6 (5, 8)	0.922	0.934 (0.924 to 0.952)
13	Q13-OAKHQOL	6 (5, 8)	6 (5, 8)	0.956	0.965 (0.856 to 0.980)
14	Q14-OAKHQOL	4 (2, 6)	4 (2, 6)	0.789	0.817 (0.791 to 0.835)
15	Q15-OAKHQOL	5 (3, 7.8)	5 (3, 8)	0.776	0.806 (0.794 to 0.838)
16	Q16-OAKHQOL	5 (1, 8)	5 (1, 8)	0.780	0.788 (0.780 to 0.842)
17	Q17-OAKHQOL	5 (4, 8)	6 (4, 9)	0.773	0.805 (0.794 to 0.847)
18	Q18-OAKHQOL	5 (1, 7)	5 (1, 7)	0.784	0.789 (0.781 to 0.889)
19	Q19-OAKHQOL	5 (3, 7.8)	5 (3, 7)	0.790	0.818 (0.792 to 0.837)
20	Q20-OAKHQOL	8 (5, 10)	8 (5, 10)	0.800	0.824 (0.798 to 0.842)

**Notes.**

Mini-OAKHQOL, Mini-Osteoarthritis Knee and Hip Quality of Life.

ICC values are <0.5, indicated as poor reliability, between 0.5–0.75 indicates moderate reliability, between 0.75 and 0.90 explains good reliability and more than 0.90 is excellent reliability.

#### Test–retest reliability

The baseline data of *n* = 208 (first session) participants who had filled out the questionnaire showed their median and interquartile range of all five dimensions as the data was not normally distributed, and in the second session (retest), *n* = 195, where thirteen participants did not turn up for the second session, hence showing a 93.75% rate of reproducibility. The test–retest reliability of the overall Arabic Mini-OAKHQOL questionnaire had an excellent ICC of 0.947 and a confidence interval (IC) of 0.774 to 0.983, as shown in Table 2. Among the five dimensions of the questionnaire, the first three showed excellent reliability, and the last two showed good test–retest reliability with a minimum time gap of one week (up to 10 days) between the two sessions, as depicted in Table 3, with their IC values and Cronbach’s alpha and intraclass correlation coefficient (ICC) of individual items shown in Table 4.

#### Factor analysis

**Figure 1:**
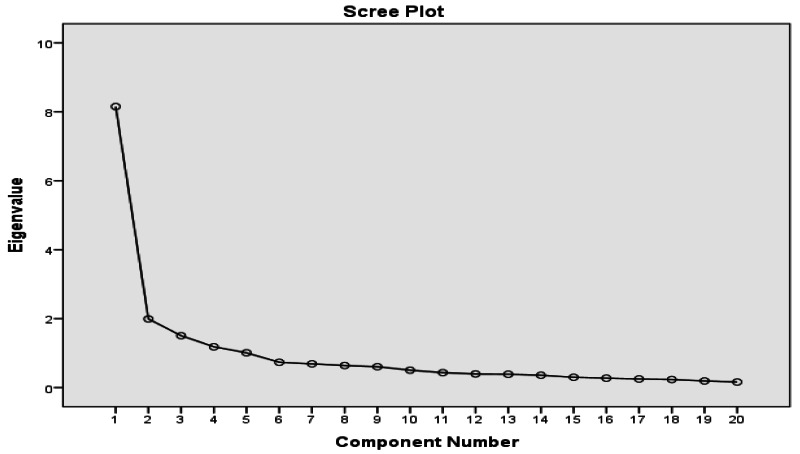
Scree plot confirming the factor number with eigenvalue (>1).

The sample’s validity (*n* = 208) was validated by a KMO measure of 0.895, which was regarded as valuable (Soriano-Alcantara, Guillén-Gámez & Ruiz-Palmero, 2024). This result showed that Bartlett’s test of sphericity (BTS) was confirmed (chi-square = 2,292.745; *p* 0). >1 and scree plot (Fig. 1), a 5-factor model with a cumulative variance of 69.212% was shown to be better able to describe the data among the 20-item Ar mini-OAKHQOL as shown in Table 5. Table 6 summarizes the factor loadings for each element in the Arabic translation of the 20 elements of Ar-Mini-OAKHQOL. We found a minimum factor loading of 0.307 and a maximum factor loading of 0.820 for this item. The factor loadings range from 0.307 to 0.820, which is acceptable for checking the validity of the factors. The overall commonality of items as shown in Table 7 varied from 0.528 to 0.781, indicating moderate commonality (Soriano-Alcantara, Guillén-Gámez & Ruiz-Palmero, 2024). The homogeneity of the 20 items of Ar-Mini-OAKHQOL was determined using principal component analysis (PCA).

**Table 5:** Five-factor model of principal component analysis. These bold item numbers 1–5 indicates: The 5-factor model of Eigenvalues with a cumulative variance of 69.212% was shown to be better able to describe the data among the 20-item Ar Mini-OAKHQOL, so these bold item values must be in bold only.

Item No.	Eigenvalues		
	Total	% Of variance	Cumulative %
1	**8.152**	**40.760**	**40.760**
2	**1.992**	**9.960**	**50.720**
3	**1.506**	**7.530**	**58.251**
4	**1.183**	**5.916**	**64.166**
5	**1.009**	**5.045**	**69.212**
6	.734	3.668	72.880
7	.690	3.448	76.327
8	.642	3.209	79.536
9	.606	3.031	82.567
10	.505	2.524	85.091
11	.433	2.164	87.255
12	.395	1.974	89.229
13	.386	1.928	91.158
14	.357	1.784	92.942
15	.299	1.496	94.438
16	.273	1.366	95.803
17	.249	1.246	97.049
18	.235	1.175	98.225
19	.194	.968	99.193
20	.161	.807	100.000

**Table 6:** Five-factor model with factor loading for 20-items.

Mini-OAKHQOL items	**5 –Factor Model**				
	**1**	**2**	**3**	**4**	**5**
Q7_OAKHQOL	.820				
Q6_OAKHQOL	.781				
Q1_OAKHQOL	.778				
Q12_OAKHQOL	.775				
Q8_OAKHQOL	.763		−.308		
Q2_OAKHQOL	.763				
Q13_OAKHQOL	.761				
Q5_OAKHQOL	.727				
Q11_OAKHQOL	.725		−.318		−.334
Q9_OAKHQOL	.709				
Q3_OAKHQOL	.681				−.442
Q10_OAKHQOL	.647				
Q16_OAKHQOL	.640		−.307		
Q4_OAKHQOL	.599		.375		.321
Q18_OAKHQOL	.549		−.408		.379
Q14_OAKHQOL		.752			
Q15_OAKHQOL		.684	.307		−.315
Q17_OAKHQOL	.351	.601			
Q19_OAKHQOL	.457		.334	−.648	
Q20_OAKHQOL		.489	.356	−.568	

**Table 7:** Overall commonality of OAKHQOL 20-items.

Ar-Mini-OAKHQOL items	Factor loading
Q1_OAKHQOL	.652
Q2_OAKHQOL	.601
Q3_OAKHQOL	.706
Q4_OAKHQOL	.664
Q5_OAKHQOL	.712
Q6_OAKHQOL	.731
Q7_OAKHQOL	.762
Q8_OAKHQOL	.687
Q9_OAKHQOL	.554
Q10_OAKHQOL	.534
Q11_OAKHQOL	.764
Q12_OAKHQOL	.761
Q13_OAKHQOL	.735
Q14_OAKHQOL	.724
Q15_OAKHQOL	.747
Q16_OAKHQOL	.528
Q17_OAKHQOL	.721
Q18_OAKHQOL	.701
Q19_OAKHQOL	.777
Q20_OAKHQOL	.781

#### Construct validity

When Ar-Mini-OAKHQOL was correlated with Ar-SF-12 physical and mental components and with VAS, it resulted in moderate to good construct validity along with overall Ar-Mini-OAKHQOL, as shown in Table 8.

**Table 8:** Construct validity of Ar-Mini-OAKHQOL with Ar-SF-12 and VAS.

Ar-Mini-OAKHQOL dimensions	Ar-SF-12 (Physical component-PCS)	Ar-SF-12 (Mental component-MCS)
Physical activity	0.713^*^	0.458^*^
Mental health	0.433^*^	0.626^*^
Pain	0.625^*^	0.530^*^
Social support	−0.313^*^	−0.304^*^
Social functioning	0.435^*^	0.461^*^
Independent items	0.231	0.212
Overall Ar-Mini-OAKHQOL	0.627^*^	0.519^*^
Overall Ar-Mini-OAKHQOL	0.578^*^	
	Pain	
	−0.537^*^	

**Notes.**

Mini-OAKHQOL, Mini-Osteoarthritis Knee and Hip Quality of Life.

* *p* < 0.001.

NS, *p* ≥ 0.05.

Spearman rank correlation was interpreted as no relationship (rs <0.25), low to fair (0.25 ≤ rs <  0.5), moderate to good (rs = 0.50–0.75), or strong (rs >  0.75).

#### Floor or ceiling effect

Tables 9 and 10 display the lowest and highest median scores for five dimensions and each Ar-Mini-OAKHQOL item, respectively. In this study, none of the independent components or dimensions exhibited a floor or ceiling effect. The values selected for the physical activity and social functioning aspects were 1.9% and 4.3%, respectively, while the values chosen for the physical activity and social support dimensions were 0.5% and 11.1%, respectively.

**Table 9:** Floor and ceiling effect of five dimensions of Ar-Mini-OAKHQOL.

Mini-OAKHQOL dimensions	Median (IQR) (25th, 75th) (*n* = 208)	Floor effect *N* (%)	Ceiling effect *N* (%)
Physical activity	47.1 (37.1, 62.9)	4 (1.9)	1 (0.5)
Mental health	50 (36.7, 70)	9 (4.2)	7 (3.4)
Pain	53.3 (36.7, 73.3)	5 (2.4)	8 (3.9)
Social support	60 (50, 80)	7 (3.4)	23 (11.1)
Social functioning	45 (25, 65)	9 (4.3)	11 (5.3)
Independent items	43.3 (24.2, 60)	18 (8.9)	1 (0.5)

**Table 10:** Floor and ceiling effects of Ar-Mini-OAKHQOL of individual items.

S No	Ar-Mini-OAKHQOL items	Median	Interquartile range (25th, 75th)	Floor effect *N* (%)	Ceiling effect *N* (%)
1	Q1-OAKHQOL	5	3, 6.8	21 (10.1)	9 (4.3)
2	Q2-OAKHQOL	6	3, 8	20 (9.6)	16 (7.7)
3	Q3-OAKHQOL	7	4, 8	20 (9.6)	25 (12.1)
4	Q4-OAKHQOL	2	0, 5	70 (13.7)	5 (2.4)
5	Q5-OAKHQOL	4	2, 6	31 (14.9)	5 (2.4)
6	Q6-OAKHQOL	4.5	2, 6	31 (14.9)	8 (3.8)
7	Q7-OAKHQOL	5	2, 7	29 (13.9)	8 (3.8)
8	Q8-OAKHQOL	6	3, 8	22 (10.6)	27 (12.9)
9	Q9-OAKHQOL	5	2, 7	38 (13.3)	16 (7.7)
10	Q10-OAKHQOL	4	0, 6	59 (12.4)	12 (5.8)
11	Q11-OAKHQOL	7	5, 9	4.8 (10)	30 (14.4)
12	Q12-OAKHQOL	6	4, 8	6 (2.9)	27 (12.9)
13	Q13-OAKHQOL	6	5, 8	7 (3.4)	13 (6.3)
14	Q14-OAKHQOL	4	2, 6	26 (12.5)	17 (8.2)
15	Q15-OAKHQOL	5	3, 7.8	23 (11.1)	31 (14.9)
16	Q16-OAKHQOL	5	1, 8	42 (12.2)	18 (8.7)
17	Q17-OAKHQOL	5	4, 8	22 (10.6)	40 (12.2)
18	Q18-OAKHQOL	5	1, 7	45 (11.6)	11 (5.3)
19	Q19-OAKHQOL	5	3, 7.8	17 (8.2)	24 (11.5)
20	Q20-OAKHQOL	8	5, 10	12 (5.8)	59 (12.4)
Overall OAKHQOL		51.4	40.1, 61.2	0 (0)	0 (0)

**Notes.**

Ar-Mini-OAKHQOL, Mini-Osteoarthritis Knee and Hip Quality of Life.

Floor and ceiling effect values are in frequencies (N) and percentage (%).

## Discussion

Translated and adapted, Ar-Mini-OAKHQOL is a practical and efficient questionnaire with cross-culturally acceptable measurement properties to measure the impact of knee or hip osteoarthritis on quality of life in all the settings of the Saudi Arabian population. The Ar-Mini-OAKHQOL retained the same five dimensions (subscales) as the original instrument after the analysis of factorial validity, which consists of 20 items: physical activities (7 items), mental health (3 items), pain (3 items), social support (2 items), and social functioning (2 items); additionally, there are three independent items that address sexual life, professional life, and fear of being dependent.

The Arabic version of Mini-OAKHQOL has an advantage with its high response rate of 93.75% during the second session and a very low attrition rate of 6.25% for the test–retest session, which authenticates that it has a surplus response rate for test and re-test reliability (Kennedy, 2022). Also, it has an advantage over the long form, as the short form has less than half (20 items) of the items of the long form (43 items), still retaining its comprehensiveness to measure the intended constructs of OA knee and hip (Guillemin et al., 2016).

It is worth discussing and comparing the results of all the original long forms of OAKHQOL with the original Mini-OAKHQOL, along with the other two mini forms that have been validated in Spanish and Turkish, to authenticate the results of the psychometric properties of Ar-Mini-OAKHQOL.

During the first stage of the translation process, grammatical errors were rectified, and a few items were rephrased for a better flow of the native language and its readability. No dissimilarities have been found in its back-translated items, which demonstrates the process of cross-cultural validation. Semantic, idiomatic, experiential (target cultural meanings), and conceptual (theoretical wordings) inequivalences were not encountered, and there were no conflicts among the translators and committee members.

The reliability of Arabic Mini-OAKHQOL in terms of internal consistency was comparable to the original long form OAKHQOL, with an excellent Cronbach’s alpha (*α*) value for the overall scale 0.931 and for individual dimensions such as physical activities (PA) 0.942, mental health (MH) 0.933, pain 0.956, and good for social support (SS) 0.782, social functioning (SF) 0.795, and independent items 0.877, which are in line with the original study dimensions such as PA, 0.96; MH, 0.92; pain, 0.91; SS, 0.81, SF, 0.73, respectively (Rat et al., 2005) and also in line with the revised original version PA 0.96, MH 0.93, pain, 0.90, SS 0.72, and SF 0.80, respectively (Rat et al., 2006). The total scale of our IC value was 0.931 and slightly better than the Moroccan Arabic long form of OAKHQOL IC value of 0.89, which demonstrates that the Ar-Mini-OAKHQOL has excellent consistency among the 20 items of the mini scale with its comprehensiveness, whereas they had discrepancies with few items and missing data (Serhier et al., 2012). When compared to the original study dimensions of Mini-OAKHQOL PA 0.95, MH 0.89, pain 0.90, SS 0.78, and SF 0.82 (Guillemin et al., 2016), our study dimensions were in line with them except that their MH had a good IC, and ours has an excellent IC. On the other hand, when compared to the mini-Spanish version obtained results, PA 0.89, MH 0.76, pain 0.81, SS 0.59, and SF 0.40, our results showed excellent to good consistency over them. According to their study, participants were eligible candidates for joint replacement, and the two social dimensions of Mini-OAKHQOL were not relevant items as with preoperative status and hence had lower outcomes (Gonzalez Sáenz De Tejada et al., 2017). The Turkish mini version IC value of PA 0.927 is in line with our value of 0.942, but the remaining dimensions were less than our IC values but still within the acceptable range (Tuncay Duruöz et al., 2022).

When compared to other long forms, the Spanish version reported good IC except for SS (Gonzalez Sáenz De Tejada et al., 2011); the Percian version has acceptable IC (Saffari et al., 2017); the Malay version has IC greater than 0.7 (Abdul Kadir et al., 2018); the Chinese version has good to excellent IC (Wang et al., 2016); and the Hungarian version has excellent to good IC (Fekete et al., 2020). The higher IC results of our study showed good to excellent OA characteristic consistency between the items of the translated Arabic version compared to all the other versions except Turkish, and our version is said to have excellent reliability for its use in the targeted Arabic male OA population.

The test–retest reproducibility of Ar Mini-OAKHQOL yielded an excellent intraclass correlation coefficient (ICC) for the first three dimensions, such as physical activities (0.968), mental health (0.947), and pain (0.965), and the rest of the two social dimensions (0.807, 0.818) had a good ICC. When compared to the original study dimensions, such as PH, 0.84; MH, 0.85; pain, 0.76; SS, 0.70; and SF, 0.70, respectively, our first three dimensions have shown excellent ICC compared to the original study, and the last two have good ICC, which are in line with the original study (Rat et al., 2005). The revised version of the original study dimensions resulted in good to moderate ICC: knee (0.80, 0.90, 0.85, 0.66, 0.59); hip (0.89, 0.83, 0.75, 0.53, 0.62), respectively (Rat et al., 2006). The ICC value of this study yielded an excellent PA dimension of 0.968 and was comparable with the Moroccan Arabic long form of OAKHQOL IC, which was 0.90; whereas Moroccan Arabic MH 0.83 and pain 0.81 were also comparable, but Moroccan SS 0.64 and SF 0.58 were less than our values as financial aid and support from family were lacking as family members had to support financially to manage OA (Serhier et al., 2012). Having self- driven vehicle habits, support from family members, and motivation for financial aid from the government, as well as frequent social gatherings in the Kingdom demonstrates that the Saudi participants with knee or hip OA have adequately responded to these two social support and functioning dimensions compared to Moroccans, and these dimensions would have also positively affected MH and pain and adequately responded to these two social support and functioning dimensions compared to Moroccans, and might have responded better than long-form of Moroccans (Serhier et al., 2012), first work (Rat et al., 2005), revised (Rat et al., 2006), and mini-original (Guillemin et al., 2016).

When compared to the original study dimensions of ICC values of 0.89, 0.86, 0.85, 0.73, and 0.66 of Mini-OAKHQOL (Guillemin et al., 2016), our study results yielded good to excellent results, and their results were moderate to good ICC (Guillemin et al., 2016). The ICC values of Spanish version PA 0.76, MH 0.74, and pain 0.74 were also comparable, but Spanish SS 0.38 and SF 0.42 were poor. They stated that after surgery, people felt less support from the world around them, even though the level of support from their illness did not change (Gonzalez Sáenz De Tejada et al., 2017).

The test–retest reliability values of Turkish versions PA 0.986; MH 0.987; pain 0.978; SS 0.843; and SF 0.962 are with excellent ICC and comparable, and slightly higher than our ICC results demonstrating these two mini scales had extremely low scale-related random measurement error (Tuncay Duruöz et al., 2022), demonstrating a reliable questionnaire to evaluate OA knee or hip impact on QoL of the population in these countries.

When compared to other long forms, the Spanish version reported good ICC except for SS and SF (Gonzalez Sáenz De Tejada et al., 2011); Percian has an acceptable ICC of Kappa 0.85 (Saffari et al., 2017); Malay has not reported ICC (Abdul Kadir et al., 2018); Chinese has a good to an excellent ICC (Wang et al., 2016); and Hungarian version has an excellent ICC in PA and a good ICC with the rest except SS and SF (Fekete et al., 2020). One possible reason for better results of all the psychometric properties in our study than the previous studies would be because the whole recruited sample were males in our study, which would have resulted in better outcomes, especially in social support and functioning dimensions due to their habit of male personal transport, social security as monthly pension schemes available to men, as well as better male outdoor activities than females in the Kingdom. Due to the higher response rate of 93.75% of our study during the second session without the influence of any treatment therapeutics, our sample would have yielded an excellent to good ICC results, indicating a consistent and reproducible questionnaire to measure QoL outcomes.

A 5-factor model with a cumulative variance of 69.212% was shown to be better able to describe the data among the 20-item Ar mini-OAKHQOL and in line with the original first and revised versions with 64% of total variance (Rat et al., 2005; Rat et al., 2006). When compared to our factor loading with the original mini-OAKHQOL, their data also showed clearly the best fit within the 5-factor model (Guillemin et al., 2016). Measuring construct validity through the factorial validity of our study is acceptable, with Malay results of the PCA, EFA, and loading analysis (Abdul Kadir et al., 2018) demonstrating our scale’s construct validity being good even when compared to different versions. Our study eigenvalue (>1) agrees with the Spanish version among all the dimensions (Gonzalez Sáenz De Tejada et al., 2017), demonstrating the unidimensional scale properties of Ar mini-OAKHQOL.

The Spearman’s rank correlation coefficient between the PA dimension of Ar Mini-OAKHQOL and the physical component (PC) of Ar SF12 showed moderate to good and low to good correlation among all the other dimensions, like the original OAKHQOL with SF 36, and also showed good to low correlation with the first and revised original versions (Rat et al., 2005; Rat et al., 2006), except for social support in both versions, where there was no correlation. Our study has a low negative correlation, and the likely negative correlation with our scale of social support would be because a decrease in physical and mental components resulted due to increased social support, and this is also a reason explaining this negative correlation: cultural variance exits with different communities (Saffari et al., 2017), like Saudi Arabians who prefer personal transport over public transport.

When five dimensions of Ar-Mini-OAKHQOL were correlated with Ar-SF-12 physical (PCS) and mental (MCS) components, the construct validity was moderate to good, along with the overall Ar-Mini-OAKHQOL. Our results showed a moderate to good positive correlation when compared to the original Mini-OAKHQOL; therefore, our results fulfil the psychometrically acceptable convergent validity. The original Mini-OAKHQOL study got a low positive correlation, but they have used a different criterion to validate construct validity with Mini-OAKHQOL, such as time since diagnosis of OA and maximal Kellgren-Lawrence (KL) score (Guillemin et al., 2016). The Moroccan version has preferred the WOMAC scale instead of SF-36, which they felt was a burden to their participants, and found a fair to strong negative correlation between OAKHQOL dimensions except for SS and the WOMAC score (a score of 96 indicates the worst symptoms). As the WOMAC score increased, OAKHQOL dimensions decreased since there was no SS in the WOMAC scale; they did not get a significant correlation, whereas with the EQ-5D, there was a low to strong positive correlation but no correlation with SS (Serhier et al., 2012).

The Spanish version of the Mini-OAKHQOL physical activity dimension showed a moderate correlation with the SF-36 physical domains (physical function *r* = 0.57; role physical *r* = 0.54, pain *r* = 0.58), but we have used Ar-SF-12. Meanwhile, the Mini-OAKHQOL pain dimension also showed a moderate correlation with the SF-36 pain dimension (*r* = 0.56). Additionally, there was a significant, moderate correlation between the mental components of the SF-36 and the Mini-OAKHQOL mental health dimension (Gonzalez Sáenz De Tejada et al., 2017).

When compared to other long forms, the Spanish version has reported a moderate correlation with SF-36 (Gonzalez Sáenz De Tejada et al., 2011); the Percian version has reported a moderate to good positive correlation with SF-12 but negative with SS. (Saffari et al., 2017); Malay has reported adequate validity with WOMAC (Abdul Kadir et al., 2018); Chinese has reported moderate to good positive correlation with SF-36 but fair with social scales (Wang et al., 2016); and the Hungarian version has reported good correlation with WHO-BREF and EQ-5D-3L (Fekete et al., 2020). And these results are supporting our results except in social dimensions where there was poverty, and the authors believe that the lack of change in the social activities component may be attributed to the old age of the participants (Gonzalez Sáenz De Tejada et al., 2017). These results match those from the original OAKHQOL questionnaire conducted by Rat et al. (2006), but our results of social support and functioning showed better outcomes as our population had better facilities in this regard, hence better correlated. The Turkish version construct validity has also shown moderate to good correlation with the SF-36 dimensions and is in line with our results except in social dimensions, as they did not get any significant correlation demonstrating the absence of the same subscale in SF-36 (Tuncay Duruöz et al., 2022), whereas we got a low to fair negative correlation indicating that as the PCH and MCH decreased, social support increased in our case. The possible explanation for this result compared to other studies would be that our populations had better facilities in terms of SS and SF in the Kingdom and documented the same outcomes.

When the VAS was correlated with the Saudi Arabic Mini-OAKHQOL, all dimensions showed a moderate negative correlation, indicating increased pain would have led to decreased physical and mental components, whereas the first original version had a good to a low correlation in all except social support, which was a non-significant correlation (Rat et al., 2005), and low to fair correlations in the revised version (Rat et al., 2006). The Moroccan version found a low to fair negative correlation between the PH, MS, and pain dimensions of OAKHQOL and VAS but found no correlation with SS and SF, as discussed earlier, having issues with SS and SF among the Moroccan population (Serhier et al., 2012), whereas we found a good positive correlation due to the well-received social dimensions by Saudis. Our VAS results are the same as the Turkish mini version for PA and pain, but MH is less than ours but not correlated with SS and SF in their study (Tuncay Duruöz et al., 2022), but our SS had a low negative correlation due to well-received social support by the family members and SF had a low positive correlation due to self-transport facilities to manage the pain during outdoor activities.

When compared to other long forms of OAKHQOL, the Spanish version reported VAS was moderately correlated (Gonzalez Sáenz De Tejada et al., 2011); the Percian version reported VAS was positively correlated with PA and MH and negatively correlated with the rest; a similar result was reported in our study (Saffari et al., 2017); and the Hungarian version reported a good correlation with VAS (Fekete et al., 2020).

The first original OAKHQOL long version has some discrimination in floor and ceiling effects (Rat et al., 2005), but obtained acceptable range values in their subsequent revised long version (Rat et al., 2006), and in the original Mini-OAKHQOL version (Guillemin et al., 2016), whereas the Moroccan version found floor and ceiling effects in a few items (Serhier et al., 2012). The Persian long version has not shown floor and ceiling effects except for the ceiling effect in SS (Saffari et al., 2017). Spanish study also did not show up any floor and ceiling effect and were in line with us except for their item no. 30 (Gonzalez Sáenz De Tejada et al., 2017); Chinese version has also reported no floor & ceiling effect (Wang et al., 2016); Malay has not reported floor and ceiling effect (Abdul Kadir et al., 2018); Turkish mini version also had floor and ceiling effect to 15% (Tuncay Duruöz et al., 2022) but this current study yielded no floor and ceiling effect neither in individual items nor in five dimensions indicating that the participants have opted for the floor and ceiling effects less than 15% in all the items representing that Ar Mini-OAKHQOL could accurately be able to measure the intended domain/construct of the OA knee and hip.

### Limitations

Due to limited time and patients were unwilling to fill out the forms several times, we did not measure the treatment responsiveness of psychometric property during this study. Only male participants were included to validate this study, as the authors had no access to female participants due to cultural barriers in the hospitals of Saudi Arabia, so the results will not be applicable or generalizable to the female population of Saudi Arabia.

## Conclusion

The transculturally adapted and tested Ar Mini-OAKHQOL is a reliable and valid questionnaire to comprehensively measure the impact of OA knee and/or hip on the quality of life of the male Saudi Arabian population.

### Future recommendations

The Arabic Mini-OAKHQOL questionnaire is required to be cross-culturally adapted and validated among Saudi Arabic females, and its responsiveness psychometric property must be validated among Saudi Arabians with OA knee and/or hip for use in research settings.

## Supplemental Information

10.7717/peerj.18122/supp-1 Supplemental Information 1 Raw data

10.7717/peerj.18122/supp-2 Supplemental Information 2 STROBE checklist

## List of abbreviations

Mini-OAKHQOL: Mini Knee and Hip Health-Related Quality of Life
OA: Osteoarthritis
QoL: Quality of life
SA: Saudi Arabia
BMI: Bodi Mass Index
SF12: Short Form 12
VAS: Visual Analog Scale
IC: Internal Consistency
ICC: Intraclass Correlation Coefficient
APC: Annual Percentage Change
HRQoL: Health-Related Quality of Life
WHO: World Health Organization
PROs: Patient-Reported Outcomes
PROMs: Patient-Reported Outcome Measures
WHOQOL: World Health Organization Quality of Life
WOMAC: Western Ontario and McMaster University
KOOS: Knee Injury and Osteoarthritis Outcome Score
ICF: International Classification of Functioning
OAQoL: Osteoarthritis Quality of Life
PHC: Primary Health Care
KSA: Kingdom of Saudi Arabia
PCS: Physical Component Score
MCS: Mental Component Score
BT1: Back Translator 1
BT2: Back Translator 2
MOH: Ministry of Health
KMO: Kaiser-Meyer-Olkin
EFA: Exploratory Factor Analysis
IC: Confidence Interval
SD: Standard Deviation
SPSS: Statistical Package for Social Sciences
Kg: Kilogram
Cm: Centimeter
BTS: Bartlett’s Test of Sphericity
PCA: Principal Component Analysis
PA: Physical Activities
MH: Mental Health
SS: Social Support
SF: Social Functioning
Vs: Verses
KL: Kellgren-Lawrence

## Additional Information and Declarations

### Data Availability

The following information was supplied regarding data availability: The raw data is available in the Supplementary File.
